# Supplementary material for: Global hypo-methylation in a proportion of glioblastoma enriched for an astrocytic signature is associated with increased invasion and altered immune landscape
Source: eLife. 2022 Nov 22;11:e77335. doi: 10.7554/eLife.77335 (PMC9681209; doi:10.7554/eLife.77335)
Supplement: Figure 2—source data 1. [file elife-77335-fig2-data1.zip › Figure_2_source_data_1/Figure_2A/homerResults/motif25.similar.html]

motif25

## Information for motif25

T
A
G
C
T
G
C
A
C
A
T
G
A
G
T
C
T
C
A
G
A
C
T
G
C
A
T
G
A
C
T
G
C
G
T
A
A
T
G
C
A
T
C
G
A
T
G
C
T
C
A
G
C
T
A
G
T
A
C
G
T
G
A
C
C
T
A
G
A
C
T
G
C
T
A
G
C
T
A
G
C
T
A
G
T
A
G
C
C
A
G
T
T
A
C
G
A
C
T
G
A
C
T
G
A
T
C
G
T
C
A
G
A
C
T
G
C
T
A
G
  
Reverse Opposite:  

G
A
T
C
T
G
A
C
A
G
T
C
T
A
G
C
A
G
T
C
G
T
A
C
A
T
G
C
G
T
C
A
A
T
C
G
G
A
T
C
G
A
T
C
G
A
T
C
G
T
A
C
G
A
T
C
A
C
T
G
A
T
G
C
G
A
T
C
A
G
T
C
T
A
C
G
T
A
G
C
T
A
C
G
G
C
A
T
T
G
A
C
G
T
A
C
G
T
A
C
A
G
T
C
T
C
A
G
G
T
A
C
A
C
G
T
A
T
C
G
  

|  |  |
| --- | --- |
| p-value: | 1e-18 |
| log p-value: | -4.188e+01 |
| Information Content per bp: | 1.546 |
| Number of Target Sequences with motif | 17.0 |
| Percentage of Target Sequences with motif | 0.73% |
| Number of Background Sequences with motif | 1.3 |
| Percentage of Background Sequences with motif | 0.04% |
| Average Position of motif in Targets | 109.2 +/- 55.0bp |
| Average Position of motif in Background | 26.0 +/- 0.0bp |
| Strand Bias (log2 ratio + to - strand density) | 0.5 |
| Multiplicity (# of sites on avg that occur together) | 1.50 |
| Motif File: | file (matrix) reverse opposite |

### Similar de novo motifs found

|  |  |  |  |  |  |  |  |
| --- | --- | --- | --- | --- | --- | --- | --- |
| Rank | Match Score | Redundant Motif | P-value | log P-value | % of Targets | % of Background | Motif file |
| 1 | 0.638 | G A T C A G T C G T A C G T A C G T A C T A C G T A G C G T A C A G T C G T A C T A G C G T A C G T A C T G A C G A T C T C G A A T G C G A C T A T G C T G C A A T G C G A T C T G A C A C T G T A G C G A T C G A T C T A G C A G T C A T G C | 1e-16 | -38.579801 | 0.69% | 0.03% | motif file (matrix) |
| 2 | 0.737 | T A C G A C G T T A C G A T C G C T G A C T A G A C T G A T G C A C T G A T C G C T A G C T A G T C A G T C A G C A T G T C A G C A T G A T C G C A T G A T C G C A T G A T C G C A T G T C A G C A G T C T A G A T C G C A T G A C T G A C T G G A T C A C T G T A C G A C T G C A T G | 1e-16 | -38.579801 | 0.69% | 0.04% | motif file (matrix) |
| 3 | 0.644 | A T G C C T G A A C T G A C G T A C T G C T A G A C T G A C T G A G C T A T G C A C G T A T G C C T A G A C T G C T A G T C G A A C G T C T A G A C T G C T A G | 1e-14 | -33.348099 | 0.78% | 0.07% | motif file (matrix) |
| 4 | 0.601 | T A C G A G C T A C G T C T A G A T C G C G A T A C T G C G A T G A T C A G T C T A C G C A G T A C T G C T A G T C A G A C T G C T A G A C T G C A T G A C T G A T C G C T G A A C T G C T A G C T A G A C G T C A T G T A C G C A T G A T C G A C T G A G T C G C A T A T C G A T C G C T A G C A T G T A C G A C T G A T C G A C T G C A T G C T G A C A T G A T C G | 1e-11 | -26.045246 | 0.52% | 0.00% | motif file (matrix) |
| 5 | 0.733 | T A G C T C G A C T A G A C G T T A C G C T A G A C T G A C T G C A G T A T G C G A T C A C T G C T A G A C T G C T A G T A C G G C A T C T A G A C T G T A C G C A T G A T C G C A G T A T C G T C A G | 1e-10 | -24.386154 | 0.73% | 0.10% | motif file (matrix) |
| 6 | 0.632 | T C G A C A T G A C T G A C T G A C T G A C G T A C T G C A T G A T C G A C T G T A G C A C G T A C T G A C T G C T G A C A T G C A T G C A T G T A C G A G C T A T C G C A T G A C T G T A G C C T A G A C G T A C T G A T C G C G A T A C T G A C T G A C T G A C T G A T G C C A T G A C T G A C T G T C G A C A T G A C T G | 1e-8 | -20.236779 | 0.43% | 0.04% | motif file (matrix) |
| 7 | 0.630 | A T G C A T G C A T G C G T A C A T G C G T A C A G T C T G A C G T A C T G A C G T A C T G A C T A C G A C T G A T G C G A T C A T G C A G C T A T C G A C T G T A G C G T A C T G A C T G A C A G T C | 1e-5 | -11.796618 | 0.39% | 0.08% | motif file (matrix) |
